# Diagnosing Patients with Age-Related Hearing Loss and Tinnitus: Supporting GP Clinical Engagement through Innovation and Pathway Redesign in Audiology Services

**DOI:** 10.1155/2012/290291

**Published:** 2012-07-08

**Authors:** Adrian Davis, Pauline A. Smith, Michelle Booth, Margaret Martin

**Affiliations:** ^1^Department of Health, Newborn and Infant Physical Examination Programme, and Newborn Hearing Screening Programme, MRC Hearing and Communication Group, Royal Free London NHS Foundation Trust, 344-354 Gray's Inn Road, London WC1X 8BP, UK; ^2^Hearing Services Department, University Hospitals of Leicester NHS Trust, Leicester LE1 5WW, UK; ^3^MRC Hearing & Communication Group, Royal Free London NHS Foundation Trust, 344-354 Gray's Inn Road, London WC1X 8BP, UK; ^4^Department of Audiology, Sherwood Forest Hospital NHS Foundation Trust. King's Mill Hospital, Mansfield Road, Sutton-In-Ashfield, Nottinghamshire NG17 4JL, UK

## Abstract

The public health challenge of hearing impairment is growing, as age is the major determinant of hearing loss. Almost one in four (22.6%) over 75-year olds reports moderate or severe worry because of hearing problems. There is a 40% comorbidity of tinnitus and balance disorders. Good outcomes depend on early presentation and appropriate referral. This paper describes how the NHS Improvement Programme in England used service improvement methodologies to identify referral pathways and tools which were most likely to make significant improvements in diagnosing hearing loss, effective referrals and better patient outcomes. An audiometric screening device was used in GP surgeries to enable thresholds for effective referrals to be measured in the surgery. Revised referral criteria, the use of this device, new “assess and fit” technology in the audiology clinic, and direct access pathways can transform audiology service delivery so that patient outcomes are measurably better. This, in turn, changes the experience of GPs, so they are more likely to refer patients who can benefit from treatment. At the end of 2011, 51 GP practices in one of the audiology pilot areas had bought HearCheck screeners, a substantial development from the 4 practices who first engaged with the pilot.

## 1. Introduction

In the UK, NHS Audiology services are complex health systems in complex environments. They provide “end-to-end” care with newborn screening, diagnostic assessment of patients, dispensing of hearing aids, and appropriate follow-up to ensure good outcomes are obtained. Historically, audiology services were commonly commissioned from the acute sector and have had a low priority because of the silent and insidious nature of the disability. In addition, the general public do not see hearing impairment as a dramatic health problem requiring urgent intervention. First presentation in the UK is usually to the General Practitioner (GP).

 Although the majority (80%) of UK patients access their hearing care through the NHS [1], there is also an option to use the independent sector without a GP referral. There are recent initiatives involving screening, for example, by telephone or internet [2], which may in future lead to self-referral without the need for GP involvement.

The public health challenge of hearing impairment is growing due to the demographics of the population, as age is the major determinant of hearing loss. Hearing impairment in the UK affects one in ten adults aged 55–74 years. Over the next 15 years hearing impairment will be an increasing population problem, because of the ageing population profile. It is likely to increase by 10–15% in population terms. Almost one in four (22.6%) over 75-year olds reports moderate or severe worry because of hearing problems [3].

Typically, those who are referred for hearing assessment have had a hearing problem for 10 or more years, are aged in their mid-70s and have a substantial hearing problem. The older that people are when they present for assessment and intervention, the more difficult they find adaptation to and care of their hearing aids. The degree of hearing impairment is a major factor that predicts ability to benefit from hearing aids. Our research for the Health Technology Assessment (HTA) Programme shows that a “disease marker” of an impairment of 35 dB hearing level (HL) at 3 k Hz gives the best outcomes for those who accept interventions. We found that 14% of the 55–74 year age group have bilateral impairment of at least 35 dB HL; only 3% had effective amplification through the use of hearing aids [3].

The HTA study also found a high comorbidity of ENT symptoms: hearing, balance and tinnitus. In 55–74 year olds, about 40% of those reporting hearing difficulties also report tinnitus, and about 20% of those reporting hearing difficulty also report both tinnitus and dizziness [3]. 

There are no research data on the number of patients with hearing loss a GP will see and refer to audiology in a year. One study in Leicester found the numbers referred in a two-year period ranged between 54 referrals from two individual GPs (with a special interest in ENT) and 1–10 patients each from 14 other individual GPs [4]. GPs will see more patients with hearing loss whom they do not refer.

GP decision-making about individual referrals is based on clinical knowledge, diagnostic tools available, knowledge of the patient, patient self-reporting, and the GP's knowledge and experience of local services and pathways. Not all patients who present to their GP with hearing problems are referred for treatment. The HTA report found that of those who have consulted their GP about hearing, only 38% also went to hospital; only 41% in the age band 55–74 years [3]. The decision not to refer is not in itself a failure and may be soundly based. More research is needed on GPs' reasons for not referring. Anecdotal evidence and information from surveys indicates that GPs may have “*low awareness of the needs of people who are deaf or hard of hearing*” [5].

The NHS Audiology Modernisation Programme, begun in 2001, has resulted in significant quality improvements, and since 2006, in reduced national waiting times. This programme also introduced provision through the independent sector for NHS patients. The major challenge recently has been high variability of referral to treatment (RTT) times, identified in 2009 through national data collection, ranging from 2 weeks direct access RTT in some audiology services to 15 weeks in others [6]. 

Another relevant policy development in the UK NHS is the introduction, through the new NHS Health and Social Care Bill [7], of “Any Qualified Provider” (AQP). Unlike the previous contractual arrangements, which were restricted to a small group of patients, this enables a variety of services to tender for and provide care to NHS patients.

### 1.1. Focus of This Study

This study focuses on two service improvement pilots that change referral criteria and pathways. The aims were:

- significant improvements in diagnosing hearing loss,
- more timely and effective referrals,
- better patient outcomes.

## 2. Materials and Methods

### 2.1. The NHS Audiology Improvement Programme 

The NHS Audiology Improvement Programme was launched in July 2009, supported by NHS Improvement [8]. The Department of Health report “Improving Access to Audiology Services” (2007) [9] included a specific commitment to apply service improvement tools. The NHS Audiology Improvement Programme recognised that a range of approaches is needed to change people's behaviour and build an evaluative culture.

### 2.2. Methods: A Range of Approaches Was Needed

 Audiology services involve a multi-stage, multi-factorial mix of human and technical processes, with often long care pathways (these are long-term conditions) and multiple human interaction variables. Using randomised controlled trials (RCTs) to develop the evidence base is not feasible for these kinds of services. Evaluative case studies, using multiple methods and different sources of evidence, were seen to be the most effective model, as the emphasis is on the real-life setting in which these services are delivered and received. 

Evidence from evaluative case studies is more likely to sustain improvements after the initial “halo effect” of pilots has gone. Ham et al. in 2002 [10] found that almost one-third of the NHS National Booked Admissions Programme pilots failed to sustain improvements. Our approach was based on staff deciding which improvements were possible in their local context, not imposing a central template.

Consequently, our hypothesis was threefold:

- evaluative case studies, using a variety of methods, would enable local staff to review their own practice,
- central and local expertise can together identify what works best. Field testing by practitioners means that it is more acceptable for national rollout,
- combining pathway redesign and peer support provides a sustainable model to build the evaluative culture needed for long term improvements.

Providing evidence of good practice from services and research is an important tool to offer commissioners: otherwise, funders will always focus on simpler targets like waiting times, numbers seen, and costs, particularly at a time when productivity and cost savings are high on the health agenda.

### 2.3. The Audiology Pilots

18 Audiology Improvement Pilot sites across all areas of audiology [11] were set up during 2009/10 to support and develop innovative ways to demonstrate measurable benefits of whole pathway redesign. They also collected qualitative data on patient-related outcomes.

The focus was on how and why these innovations succeed or fail, and how the context in which they are delivered would influence the outcome. The immediate challenge was how could busy staff engage with change and innovation whilst at the same time keeping the “everyday business” of audiology services running? This paper describes two of the 18 pilot projects, both of which involve GP decision making and direct referral to audiology.

These two pilots focused on age-related hearing loss and tinnitus, in particular, improving primary care referrals by supporting greater GP clinical engagement. These examples focus on those critical interfaces where, historically, the hearing care journey can so easily break down for patients: between primary and secondary care, particularly for patients with tinnitus and complex hearing needs.

The work relies on moderately small numbers, but the results are sufficiently robust to indicate the scale of improvements which may be possible. Findings are likely to become more substantial when greater numbers of patients are treated as the pilots move into mainstream provision.

#### 2.3.1. Triage in Primary Care: University Hospitals of Leicester NHS Trust

The traditional way to manage patients who may need a hearing aid is for the GP to refer to the audiology service: the patient is assessed at one appointment and fitted with aid/s at another, after the custom made earmould/s have been manufactured. However, recent advances in technology, specifically “open fit” hearing aids [12] mean that for some patients, these two appointments can be combined and suitable patients assessed and fitted (A&F) at the same appointment. Open fit hearing aids have been shown to provide increased comfort to patients, and a more natural quality of sound, as well as being more discreet. One appointment saves time, so that if suitable patients can be identified before they arrive at audiology (i.e., if they are triaged in primary care), then the process can become very efficient for both patients and service providers.

This project involved devising a new referral form for GPs, which together with a very simple hearing screening device, allowed the audiology service to direct patients either to the traditional two-appointment pathway or the new assess and fit pathway. 

The overall aim of the Leicester pilot, which ran during 2009 and 2010, was to promote GP clinical engagement locally by enabling GPs to triage their patients. The project also provided GPs with up-to-date information about hearing aid technology and hearing services.

The hearing screening device that was used had been developed by Siemens and the MRC Hearing & Communication Group. The *HearCheck Screener *[13] can identify age-related hearing problems and predict people who could benefit from intervention. It is a simple, low-cost, hand-held device which produces a fixed series of 6 pure tones, (75, 55, and 35 dB HL at 3 kHz and 55, 35, and 20 dB HL at 1 kHz). It is unable to identify low frequency or conductive hearing loss, so needed to be combined with providing GPs with revised referral criteria and information on management of hearing loss. Better information has emerged as one of the key successes of the pilot. This meant that GPs were also then able to brief patients on the nature of their condition, telling them that they may get a hearing aid at one appointment if that were suitable, so their patients were clear what to expect at each stage of their pathway.

At the start of the project the audiology team worked with four GPs. These four later involved all their GP partners. At the end of the project the audiology team were working with 11 practices. Two of the four original practices used health care assistants to carry out the triage on their behalf. The engagement, interest, and support of the four GP practices involved in the initial pilot of the technology and questionnaire were integral to its success.

The screening devices, which cost around *£*100 each, were loaned to the GPs in the pilot. Calibration is needed every three years and the devices are expected to last around five years. The audiology team recorded all details of the triage, and all subsequent measurements and outcomes for every patient, (including qualitative data) to enable a full analysis of the triage in terms of the agreed outcome measures.

#### 2.3.2. Direct Access Tinnitus Pathway: Sherwood Forest Hospitals NHS Trust

In Nottinghamshire, patients with tinnitus were being referred via ENT; appropriate patients would then be referred onto audiology for specialist advice and support, which added an extra step and wait into the process. A local audit found a high rate of non-responders for patients referred from ENT, and significant waits and bottlenecks in the referral pathways for tinnitus patients.

The aim was to pilot direct access from primary care for patients with tinnitus. This was also an opportunity to provide information about the condition and specialist support available.

All local GPs were able to refer into the service to maximise the numbers and allow for a measurable evaluation of the outcomes. Clear referral criteria were drawn up for GPs to ensure appropriate patients would be referred. The clinic was set up on the Choose and Book System to allow referrals to be accepted electronically. GPs were also notified of the service via the Trust's GP bulletin. A press release was also issued to raise public awareness to coincide with National Tinnitus Week.

Data on all waiting times were recorded and a detailed patient satisfaction questionnaire was completed by all directly referred tinnitus patients. Numbers of patients requiring or requesting subsequent ENT consultations were recorded to ensure that all aspects of an efficient service were considered.

## 3. Results

### 3.1. Hearing Triage in Primary Care: University Hospitals of Leicester NHS Trust

At the end of the trial, 97 patients were referred who had been triaged in primary care for age, hearing, vision, ability to concentrate and manual dexterity. 53 not suitable for A&F were invited for the traditional pathway (23 excluded on age, 39 excluded on hearing, 1 on vision, 1 on cognition, 0 on manual dexterity). 44 patients suitable for A&F were invited for 90-minute appointments. 5 opted for different appointments or cancelled, so 39 attended the clinic. 26 were fitted on the day, 4 at a later date, and 9 had hearing too good for, or declined, a hearing aid. This equates to 27% of referred patients being A&F in a single appointment. The work relies on moderately small numbers, so some caution in interpretation is needed, but the numbers are sufficient to justify some rough estimates of savings which could be made. For example, in the last 12 months, the department received 2600 direct referrals, so if 27% of them had been A&F, the number of A&F would be 702 patients, giving a time saving per year of 350 clinical hours.

**Patient-Related Outcome Measures**

Patient related outcome measures demonstrated that the quality of service had not been compromised by the A&F procedure. There was a good correlation between the primary care screening test result and the clinic results, indicating that the HearCheck is providing the right information for triage. Importantly, the pilot also found that the patients were more likely to be referred at their first visit. Data from the patient satisfaction questionnaire found that 84% respondents said that their GP referred them without delay, ensuring that the management of their hearing loss could be promptly assessed.

By the end of 2011, a further 51 GP practices had bought HearCheck screeners, a substantial development from the 4 practices who first engaged with the pilot. Significantly, it is also the GPs themselves who are “spreading the word.” They had seen the benefits and were able to secure meetings for the audiology team with other practices.

### 3.2. Direct Access Tinnitus Pathway: Sherwood Forest Hospitals NHS Trust

The pilot ran from December 2009 to December 2010 and 100 patients were seen through the new pathway. During the pilot the average waiting time referral to treatment (RTT) for tinnitus patients was reduced from 14 weeks (range = 5 to 53 weeks) to 2 weeks (range = 1 to 9 weeks). 23% (23 out of the 100 patients seen) were “red flagged” as requiring/requesting referral to ENT and 43% were seen as “one stop service.” The quality of referrals received from GPs was high, and few inappropriate referrals were received.

**Patient-Related Outcome Measures**

The patient satisfaction questionnaire found that

- 100% of patients felt their first hospital appointment was sooner than they had thought it would be,
- 86% felt more confident to manage their condition better,
- 86% had left their appointment with an understanding of the plan for their care.

### 3.3. Barriers and Challenges in the Pilots

Time was a constant challenge. Stakeholder and team engagement were sometimes difficult, because of busy workloads and the need to change mindsets. Creating opportunities to inform GPs about present day audiology services was challenging. The initial cost of the HearCheck screener could prove a sticking point with some GPs but it can be argued that for the long term, savings in the reduction of the patient pathway will cover these concerns.

## 4. Discussion

These pilots demonstrate that engaging GPs in service improvement is most successful when they are *clinically *engaged, by providing information on management of hearing loss, up-to-date information on local pathways, clear referral criteria, and, where available, the use of innovative technology like the HearCheck screening device. The results show significant improvements in referral patterns and benefits for patients.

On-going communication with GPs at every opportunity has been integral to the success and sustainability of these pilots, which were always viewed as longer-term projects. By the end of 2011, a further 51 GP practices in the Leicester pilot area had bought HearCheck screeners.

We now have new tools and pathways to support initial assessment of hearing loss in primary care. As services improve, and patients who have been referred report good experience and outcomes back to their GP, so GPs' internal map of local services will change and so will their referral patterns. When audiology staff work with GPs to provide tools for referring patients more effectively, the resulting service benefit will help re-draw that internal map more quickly. Throwing a “spotlight” in this way on the pattern of care patients receive in a service which has low priority in the NHS shows how changing the pathway can improve the service, leading in turn to better models for commissioning of audiology services in the future.

A national publication, “Pushing the Boundaries,” has been produced by NHS Improvement to share the learning from the pilot phase of the programme by describing in detail the work in the 18 pilot sites [4]. A second publication “Shaping the future: Strengthening the evidence to transform audiology services” [14] was published in March 2011.

As the NHS moves into a new phase involving new commissioning models and potential new providers through the AQP route, new possibilities will continue to emerge. Future pathways will need to reflect the development of new technologies, enabling self-assessment, for example, through internet or telephone platforms, which could lead to self-referral to audiology services.

## 5. Conclusions

GPs have a critically important role in the current hearing care pathway: appropriate and early referral is essential.

Improved referral criteria based on research and better information on hearing loss for GPs, and a simple triage using an audiometric screening device, can lead to earlier identification and management of hearing problems in primary care. People with hearing impairment are highly likely to have other problems such as tinnitus and balance disorders. There is a 40% comorbidity. Balance disorders contribute in part as risk factors for falls and other accidental injury which are frequent causes of loss of independence, avoidable illness and mortality. Given the comorbidity, there is an added opportunity for GPs to improve benefit for their patients, if tinnitus and dizziness could also be identified and managed.
